# Chemometric-assisted QuEChERS extraction method for post-harvest pesticide determination in fruits and vegetables

**DOI:** 10.1038/srep42489

**Published:** 2017-02-22

**Authors:** Minmin Li, Chao Dai, Fengzhong Wang, Zhiqiang Kong, Yan He, Ya Tao Huang, Bei Fan

**Affiliations:** 1 Institute of Food Science and Technology, Chinese Academy of Agricultural Sciences/Key Laboratory of Agro-Products Processing/Laboratory of Agro-Products Quality Safety Risk Assessment, Ministry of Agriculture, Beijing 100193, P.R. China; 2 Functional and Evolutionary Entomology, Gembloux Agro-Bio-Tech, University of Liège, Passage des Déportés 2, 5030 Gembloux, Belgium

## Abstract

An effective analysis method was developed based on a chemometric tool for the simultaneous quantification of five different post-harvest pesticides (2,4-dichlorophenoxyacetic acid (2,4-D), carbendazim, thiabendazole, iprodione, and prochloraz) in fruits and vegetables. In the modified QuEChERS (quick, easy, cheap, effective, rugged and safe) method, the factors and responses for optimization of the extraction and cleanup analyses were compared using the Plackett–Burman (P–B) screening design. Furthermore, the significant factors (toluene percentage, hydrochloric acid (HCl) percentage, and graphitized carbon black (GCB) amount) were optimized using a central composite design (CCD) combined with Derringer’s desirability function (DF). The limits of quantification (LOQs) were estimated to be 1.0 μg/kg for 2,4-D, carbendazim, thiabendazole, and prochloraz, and 1.5 μg/kg for iprodione in food matrices. The mean recoveries were in the range of 70.4–113.9% with relative standard deviations (RSDs) of less than 16.9% at three spiking levels. The measurement uncertainty of the analytical method was determined using the bottom-up approach, which yielded an average value of 7.6%. Carbendazim was most frequently found in real samples analyzed using the developed method. Consequently, the analytical method can serve as an advantageous and rapid tool for determination of five preservative pesticides in fruits and vegetables.

Over the last few decades, there has been a worldwide trend toward the consumption of more vegetables and fruits, as they are important sources of vitamins and fiber, contributing to a healthy lifestyle and prevention of diseases^1^. Owing to the obvious effects of sterilization and antisepsis, preservative pesticides are largely applied to fruits and vegetables from post-harvest to storage or long distance transport; however, there is a risk that toxic residues from the applied pesticides will be accumulated in foodstuffs. The most popular post-harvest pesticides in developing countries include 2,4-dichlorophenoxyacetic acid (2,4-D), carbendazim, thiabendazole, iprodione, and prochloraz^2^,^3^, which are also widely used in agricultural practices. In particular, 2,4-D is widely used in Chinese agriculture to eliminate weeds in crops.

Regulations have established legal maximum residue levels (MRLs) for these pesticides in fruits and vegetables. Using citrus as an example, the MRLs in citrus fruits (such as oranges) in China for 2,4-D, carbendazim, thiabendazole, and prochloraz are 1.0, 5.0, 10.0, and 10.0 mg/kg, respectively; however, no MRL has been established for iprodione in citrus fruits^4^. Under the European Union (EU) regulation (EC) No. 396/2005, the MRLs in oranges for 2,4-D, carbendazim, thiabendazole, iprodione, and prochloraz are 1.0, 0.2, 5.0, 0.01, and 10.0 mg/kg, respectively^5^. The European Food Safety Authority’s (EFSA) annual report for 2013 showed that 2,4-D, carbendazim, thiabendazole, iprodione, and prochloraz residues were detected at or below the MRL in 0.0%, 2.6%, 0.72%, 6.8%, and 0.03% of plant products analyzed, respectively. Moreover, MRL exceedances were most frequently recorded for carbendazim (0.2%)^6^. Furthermore, one of the most frequently detected pesticides in orange samples was thiabendazole (25.9%)^7^. Therefore, monitoring of these pesticides in fruits and vegetables is important to ensure food safety.

Some methods for the individual determination of 2,4-D, carbendazim, thiabendazole, iprodione, and prochloraz in food matrices have been previously reported using gas chromatography coupled to mass spectrometry (GC–MS)^8^,^9^, high-performance liquid chromatography (HPLC)^10^,^11^, and liquid chromatography coupled to tandem mass spectrometry (LC–MS)^3^,^12^. However, simultaneous determination of these five compounds in food samples is currently not available. In particular, as the physicochemical properties of 2,4-D differ from those of many other pesticides, owing to its highly solubility in water and high melting point^13^,^14^, simultaneous determination of pesticide residues with 2,4-D often proves difficult. Liquid chromatography coupled with tandem mass spectrometry (LC–MS/MS) has proved to be a powerful and widely used technique for the analysis of pesticides at trace concentration levels because of its high selectivity, precision, and sensitivity^14^. Despite these advantages of LC-MS/MS, an important drawback of electrospray ionization that has been considered more frequently in recent years is the matrix effect. The matrix effect can severely compromise the quantitative analysis of trace-level compounds, as well as method reproducibility^15^. Various approaches have been proposed for minimizing or eliminating the matrix effect, such as improving chromatographic selectivity to avoid coelution of compounds and matrix components^16^, using different mobile phase strengths or modifiers^17^, and modifying sample preparation procedures to remove interferences^18^. Dilution is an easy and effective method to remove interfering compounds, and the development of new-generation commercial analytical instruments with high sensitivity makes this approach feasible^15^,^19^.

The QuEChERS (quick, easy, cheap, effective, rugged and safe) method, which was developed by Anastassiades *et al*.^20^, has proved to be an attractive pretreatment method for pesticide multiresidue analysis in fruits and vegetables. Nevertheless, in many analytical methods, the importance of interactions between factors is often not taken into account. Hence, conventional optimization strategies for analytical methods often fail to achieve exact specifications. Chemometrics applies four main techniques, including screening, optimization, time-saving, and quantitation, to analytical methods^21^, with some limitations^22^. A Plackett-Burman (P–B) experimental design is used to identify the most important factors early in the experimentation phase when complete knowledge about the system is usually unavailable^23^. Developed in 1946 by statisticians Robin L. Plackett and J.P. Burman^24^, it is one of most widely used chemometric methods used for screening of factors because it is both economic and efficient^25^. The P–B design methodology is a powerful and practical tool for rapidly determining key variables in a multivariable system^26^. Central composite design (CCD) combines a two-level factorial design with a star design and centre points. The star and factorial points can lie equidistant from the centre, or the star points can lie within the space of the factorial design or they can lie on the faces of the factorial design points^27^. The use of CCD allowed the determination of the levels of various parameters to be carried out with simultaneous evolution of the interrelation between each parameter^28^. This method has been successfully applied in the optimization of medium composition^29^. The desirability function approach is one of the most widely used methods in industry for the optimization of multiple response processes, and the useful class of desirability functions was proposed by Derringer and Suich^30^. In addition, the so-called “Derringer’s desirability function” (DF) is a powerful strategy for simultaneous optimization of different objective functions (responses)^25^,^31^.

In this study, the chemometric methods including P–B design, CCD, and DF statistical techniques were used to modify QuEChERS method for the analysis of 2,4-D, carbendazim, thiabendazole, iprodione, and prochloraz in fruits and vegetables using ultra high performance liquid chromatography coupled with tandem mass spectrometry (UHPLC–MS/MS), and sample dilution was investigated to diminish the matrix effect. Moreover, the effectiveness and applicability of the developed method were evaluated in real samples.

## Results and Discussion

### Optimization of chromatographic and MS/MS conditions

To ensure a satisfactory chromatographic separation of the five studied pesticides, a series of experiments were carried out with different columns (Agilent ZORBAX SB-C18, Poroshell120 SB-C18, and Poroshell120 EC-C18 columns), to improve the peak shape and resolution from the interfering and noise peaks. The Poroshell 120 EC-C18 (2.1 × 50 mm, 2.7 μm) column were selected as it showed higher efficiency and a shorter equilibrium time compared with the other columns, which may be due to the inner solid core and porous silica outer layer applied to the EC-C18 bonded phase^32^,^33^. Various mobile phase compositions employed in reversed phase chromatography and electrospray ionisation (ESI) methods (i.e., water–acetonitrile and water–methanol with different concentrations of formic acid and ammonium formate added to the aqueous phase) were investigated using the gradient program with a 0.4 mL min^−1^ flow rate. Higher sensitivity with good peak shape was attained when water–methanol was used without any formic acid or ammonium formate. Although, formic acid in water improves the formation of protonated adducts, it can inhibit the negative ESI mode during UHPLC–ESI-MS/MS analysis. As shown in Supplementary Figure 1, there was no interference at the retention times of the analytes, and the analysis time for the five pesticides was less than 5.0 min. The compounds were eluted in the following order: carbendazim (1.218 min), prochloraz (1.371 min), 2,4-D (2.688 min), thiabendazole (4.041), and iprodione (4.941 min).

In this study, the multi-reaction monitoring (MRM) mode was used to perform the analysis, and the five target compounds presented comparable ionization in both positive and negative modes. ESI in positive mode was selected for the determination of carbendazim, thiabendazole, iprodione, and prochloraz, as somewhat higher responses were obtained, whereas the response signal for 2,4-D was higher in the negative mode. All of the compounds had abundant [M + H]^+^ ions ([M − H]^−^ ions for 2,4-D), which were usually selected as the precursor ions. According to the European Commission Decision 2002/657/EC^34^, confirmation and identification is based on the accumulation of identification points (IPs). The spectrum derived from a LC-MS/MS method achieves four IPs (1.0 IP for the precursor ion, and 1.5 IP for each of the two product ions), which allows the identity of most compounds to be confirmed. Identification was conducted based on the retention time, the two selected ion transitions, and their relative abundance. The molecular weights, precursor ions, product ions, fragmentor voltages, and collision energies for the five analytes are listed in Supplementary Table 1.

### Optimization of sample pretreatment procedure

The QuEChERS procedure is the combination of an extraction step for pesticides in fruits and vegetables and a cleanup step that removes sugars, lipids, and organic acids. During these two steps, many factors that can affect the extraction efficiency. To evaluate and optimize the parameters that affect the QuEChERS procedure, a screening design (P–B design) was used to determine the significant factors and an optimization design (CCD) was used to estimate the best experimental conditions.

### Screening design

**Figure 1:**
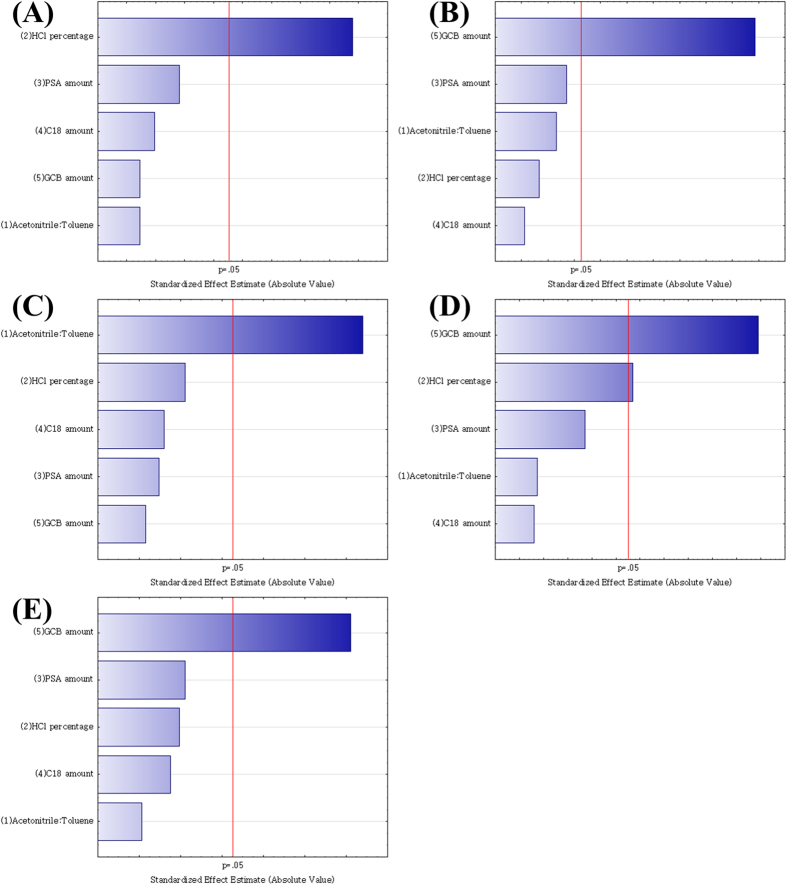
Standardized Pareto charts obtained from the Plackett–Burman design. (**A**) 2,4-D, (**B**) carbendazim, (**C**) thiabendazole, (**D**) iprodione, and (**E**) prochloraz.

In this work, the P–B design was generated to screen the most important factors that affect the QuEChERS efficiency and the recovery of the five pesticide residues. As 2,4-D is a relatively strong acid (pKa = 3) and more stable at low pH values^35^, it is important to maintain pH control in the extraction solvent. Moreover, as the dissociated form of 2,4-D is highly polar, it is soluble in aqueous solutions and less soluble in water-immiscible organic solvents^36^, whereas carbendazim, thiabendazole, iprodione, and prochloraz are readily soluble in most organic solvents (i.e., methanol, acetonitrile, and acetone). Therefore, the addition of toluene to the extraction solvent was examined to improve the recoveries. In this study, five factors, namely, the extraction solution composition (i.e., toluene percentage, X_1_, 0–100%), HCl percentage in the extraction solution (X_2_, 0–0.5%), primary secondary amine (PSA) amount (X_3_, 0–50 mg), octadecylsilane (C18) amount (X_4_, 0–20 mg), and graphitized carbon black (GCB) amount (X_5_, 0–20 mg) were studied (Supplementary Table 2). The main effect of each factor was investigated in 15 runs (12 + 3 center points), and analysis of variance (ANOVA) and a *t*-test at a 95% confidence level were employed^37^. To reduce the effect of uncontrolled variables, the P–B experiments were run in a random manner. The effects of the factors in the P–B design are illustrated in a standardized Pareto chart (Fig. 1); the length of the bar is proportional to the absolute value of the main effect, while the vertical line indicates the 95% confidence level. As illustrated in Fig. 1, the GCB amount was the most significant variable, yielding a negative effect for all target analytes, except 2,4-D and thiabendazole. The percentage of HCl was the next most significant variable, followed by the percentage of toluene, and these variables exerted a positive effect. Therefore, for the optimization step, all other factors were fixed, while the GCB amount, percentage of HCl, and percentage of toluene were considered for further optimization.

### Optimization design

**Figure 2:**
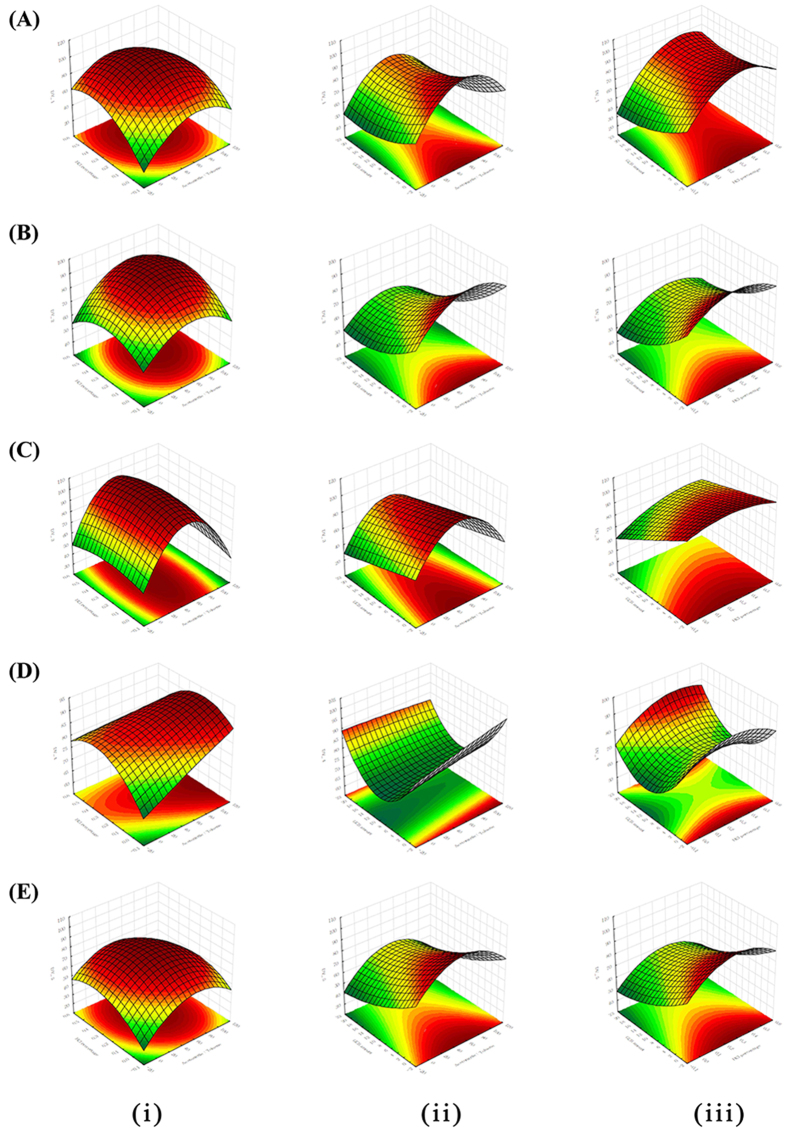
Response surfaces of the recoveries of (**A**) 2,4-D, (**B**) carbendazim, (**C**) thiabendazole, (**D**) iprodione, and (**E**) prochloraz estimated from the central composite design by plotting the (i) toluene percentage (%) versus HCl percentage (%), (ii) toluene percentage (%) versus GCB amount (g), and (iii) and HCl percentage (%) versus GCB amount (g).

The screening experiment obtained using the P–B design indicated that the PSA amount and C18 amount do not affect the extraction efficiency to any significant extent. Therefore, they were eliminated from further studies. The GCB amount, percentage of HCl, and percentage of toluene, which are the significant variables, were further optimized using second-order CCD with a response surface methodology. ANOVA for the response surface model was carried out to assess the accuracy and quality of the fitted model using the coefficient of determination (R^2^) values. The regression analysis results indicated that the quadratic model contribution was statistically significant (p < 0.05). The lack-of-fit (LOF) test was not significant (p > 0.05), demonstrating that the model fitted the response well. R^2^ values of 0.9659, 0.9331, 0.9447, 0.8478, and 0.9380 were obtained for 2,4-D, carbendazim, thiabendazole, iprodione, and prochloraz, respectively, which indicated that the fitted models were adequate to describe the relationship between the response and the variables. The regression coefficients and the probability values of each variable in the model are shown in Supplementary Table 3. The percentage of toluene (X_1_) and the GCB amount (X_3_) had the most significant effects on the extraction yields at the 95% confidence level, with the exception of iprodione and thiabendazole, respectively. The HCl percentage (X_2_) only affected the recoveries of 2,4-D, iprodione, and prochloraz. Among the quadratic terms, X_1_^2^ was significant for 2,4-D, thiabendazole, and prochloraz, whereas X_2_^2^ and X_3_^2^ were only significant for prochloraz and iprodione, respectively. The interaction terms were not significant for any of the responses, with the exception of X_1_ X _2_ and X_2_ X _3_ for 2,4-D. To evaluate the trends in toluene percentage, HCl percentage, and GCB amount, three-dimensional (3D) response surface plots for the five analytes were constructed, as shown in Fig. 2.

**Figure 3:**
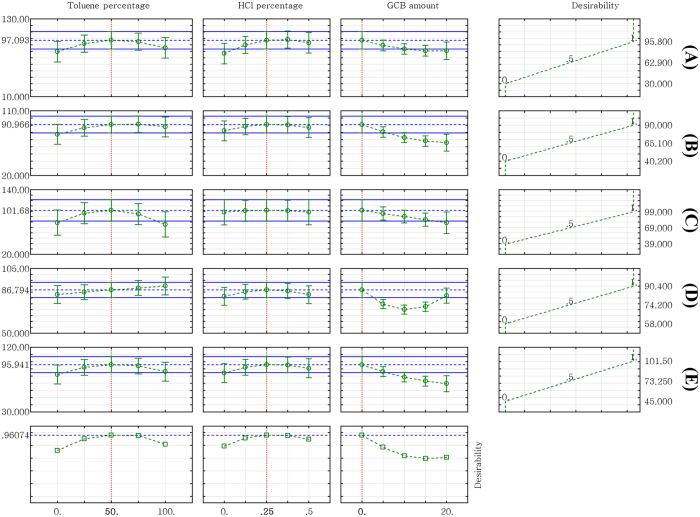
Profiles of predicated values and desirability functions for the extraction recovery of (**A**) 2,4-D, (**B**) carbendazim, (**C**) thiabendazole, (**D**) iprodione, and (**E**) prochloraz. The dashed lines indicate the values after optimization.

The desirability profiles obtained from the predicted values using the Statistica 10.0 software were used for the optimization process. The scale in the range of 0.0 (undesirable) to 1.0 (very desirable) should be maximized by efficient selection and optimization of the variables. The CCD optimization design matrix (Fig. 3) shows that the maximum recoveries of 2,4-D (95.8% with a desirability of 1.0), carbendazim (90.0% with a desirability of 1.0), thiabendazole (99.0% with a desirability of 1.0), iprodione (90.4% with a desirability of 1.0), and prochloraz (101.5% with a desirability of 1.0) were achieved under the following conditions: extraction solvent of 1:1 acetonitrile:toluene (v/v) containing 0.25% HCl and 0 mg GCB.

### Method validation

The method was validated in accordance with the SANCO/12571/2013^38^, which is a method validation procedure for pesticide residue analysis in food that includes the following parameters: accuracy, precision, linearity, matrix effects, and limit of quantifications (LOQs).

### Linearity

Linearity was evaluated using standard solutions, which were diluted using methanol, and matrix-matched calibration curves for eight blank sample extracts (citrus, apple, mango, lychee, tomato, cucumber, green pepper, and eggplant) with concentration gradients of 0.1, 1, 5, 10, 50, 100, and 200 μg/L for 2,4-D, carbendazim, thiabendazole, and prochloraz, and 0.25, 1, 5, 10, 50, 100, and 200 μg/L for iprodione. The calibration method greatly influences the quantitative determination results. Good linearity was observed for all the target pesticides with R^2^ values greater than 0.9900 for the blank extracts and the pure solvent-based solutions without dilution and with 10-fold dilution (0.9940–0.9999).

### Matrix effect

When using ESI, the presence of matrix components can affect the ionization of the target compounds^39^. The matrix effect was detected by comparing the slopes of the calibration curves for the blank sample extracts (without dilution and with 10-fold dilution) with those for pure solvent. Signal suppression or enhancement can seriously compromise quantitation of a target compound at trace levels, and greatly affect the reproducibility and accuracy of the method^15^. Signal enhancement occurs if the percentage difference between the slopes of the calibration curves is positive, whereas if the difference is negative, signal suppression occurs. The magnitude of this percentage indicates the extent of the matrix effect. No matrix effect is considered to occur when the value is between −20% and 20% because this variation is similar to the repeatability values. However, values below −50% or above 50% are considered to correspond to strong matrix effects, and others are recognized as medium matrix effects.

**Figure 4:**
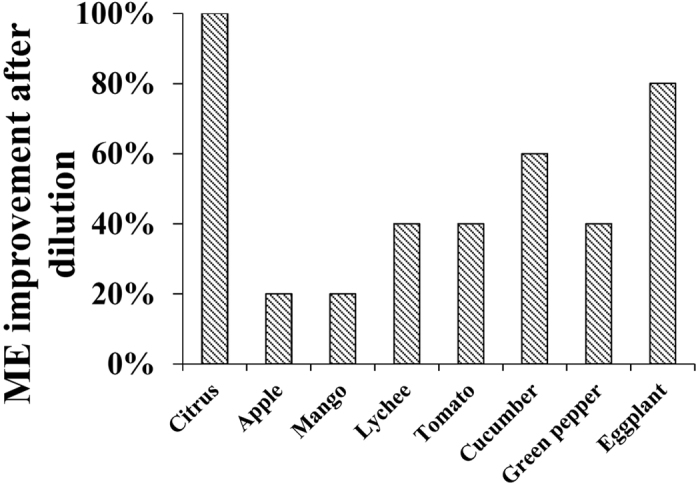
Improvement of the matrix effect in the eight matrices after 10-fold dilution.

For the extracts without dilution, 2,4-D, carbendazim, and thiabendazole in citrus, carbendazim in cucumber, thiabendazole and iprodione in lychee, and iprodione in eggplant exhibited strong matrix effects. This is because of the complexity of the interfering compounds in citrus, cucumber, lychee, and eggplant matrices. Using LC-Q-TOF-MS, Ferrer *et al*.^15^ identified one interfering compound as nobiletin, which was mainly present in citrus peel. The dilution of the sample extracts with pure solvent was assayed to examine signal suppression following reduction of the matrix load. As shown in Fig. 4, the matrix effect of citrus and eggplant improved 100% and 80%, respectively, after 10-fold dilution. Moreover, more than 20% improvement was obtained for the other samples. Meanwhile, each pesticide showed completely different behavior, an illustrative example of which is thiabendazole (Table 1). In citrus or in lychee, thiabendazole shows high signal suppression or enhancement, but the matrix effect was significantly decreased with dilution; however, even without dilution, the matrix effect in apple is negligible. Some pesticides will interact with complex components of the matrix sample at very low concentrations, resulting in signal suppression, even though the extracts are highly diluted. As the average signal for some pesticides after dilution was still half that of the solvent standards, matrix-matched calibration was required using blank extracts diluted 10-fold with methanol.

### Limits of quantification and recovery study

The LOQs were determined according to the lowest concentration level validated (1.0 μg/kg for 2,4-D, carbendazim, thiabendazole, and prochloraz, and 1.5 μg/kg for iprodione) in food matrices with satisfactory recoveries of between 70% and 120% and. relative standard deviations (RSDs) of less than 20%. The recovery (trueness and precision) and repeatability (intra-day and inter-day) of the described method were determined in spiked blanks at three concentration levels (LOQ, 10 × LOQ, and 100 × LOQ) in five replications. Excellent average recoveries in the range of 70.4–113.9% were obtained at all spiking levels. Moreover, good repeatability with intra-day (n = 5) and inter-day (n = 15) RSDs for the proposed method ranging from 0.6 to 11.9% and from 1.2 to 16.9%, respectively, were also obtained (Table 1). The recovery assay results illustrate that this method has good precision and accuracy for all five compounds analyzed in citrus, apple, mango, lychee, tomato, cucumber, green pepper, and eggplant.

### Uncertainty

The uncertainty associated with an analytical methodology describes the range around a reported or experimental result within which the true value can be expected to lie with a defined level of probability^40^. In this study, the measurement uncertainty was determined for all compounds at three spiked levels using the bottom-up approach based on the in-house validation data, in accordance with EURACHEM/CITAC^41^. The main sources of uncertainty were identified and quantified, and the combined uncertainty (*U*_c_) was calculated as follows:


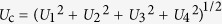


Uncertainty *U*_1_, which is associated with the preparation of standards and stock solutions, is concentration-dependent and was calculated by the propagation of errors approach. Uncertainty *U*_2_, which is associated with the calibration curve, represents the contribution of estimating the analyte concentration from the calibration curve. Uncertainty *U*_3_, which is associated with the precision, is expressed as the RSD obtained from repeatability or intermediate precision assays for different concentration levels. Uncertainty *U*_4_, which is associated with the accuracy, is the recovery percentage obtained from recovery assays. The expanded uncertainty (*U*_exp_) was obtained from the combined uncertainty by multiplying by a coverage factor *k* = 2 to ensure a level of confidence of 95%, as follows:


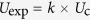


The results obtained for each individual source of uncertainty, the combined uncertainty *U*_c_, and the expanded uncertainty *U*_exp_ are summarized in Table 2. The *U*_exp_ values were 8.5%, 5.9%, 7.7%, 7.5%, and 8.4% for 2,4-D, carbendazim, thiabendazole, iprodione, and prochloraz, respectively, which yielded an average value of 7.6%. This uncertainty is distinctly lower than the maximum threshold value of 50% recommended by SANCO/12571/2013^38^, which clearly demonstrates the fitness for purpose of the developed method.

### Monitoring and safety evaluation of market samples

The effectiveness and applicability of this method for measuring trace levels of the target compounds were evaluated by randomly analyzing 85 real samples (20 citrus, 10 apple, 10 mango, 20 lychee, 5 tomato, 5 cucumber, 10 green pepper, and 5 eggplant samples) obtained from different local markets in Beijing (China). The determined concentrations of detected pesticides (Table 3) show that 88% of the samples were blank or contained pesticides at levels lower than the LOQs, while 12% of the samples contained one or more of the pesticides studied. Three different pesticides were detected in some of these samples, and carbendazim was most commonly found in the samples. The highest pesticide residue concentration was found for carbendazim in citrus at 12.8 μg/kg. Moreover, citrus had the highest positive sample ratio for detected pesticide residues, mainly containing carbendazim, thiabendazole, and 2,4-D. These results are in agreement with previous literature reports, in which the majority of orange samples analyzed contained these pesticide residues^14^,^15^. It is important to note that all detected pesticides were below the MRLs established by Chinese and European MRL regulations^4^,^5^. Hence, the presence of these pesticides at these levels in some of the samples does not pose a threat to the consumer.

## Conclusion

An effective method for the simultaneous quantification of 2,4-D, carbendazim, thiabendazole, iprodione, and prochloraz in fruits and vegetables was developed using QuEChERS and UHPLC–MS/MS. The extraction and cleanup steps of the QuEChERS method were optimized using chemometrics, with the significant factors determined using a P–B screening design and subsequently optimized using CCD combined with DF. The optimum extraction solution consisted of acetonitrile:toluene (1:1, v/v) with 0.25% HCl and 0 mg GCB. The develop method was validated with good accuracy, linearity, LOQs, recoveries, and measurement uncertainty. Matrix-matched calibration was required to compensate for matrix effects. The successful application of the developed method to real samples confirmed its reliability and efficacy for routine pesticide residue monitoring in vegetable and fruit samples.

## Materials and Methods

### Reagents and materials

Analytical standards of 2,4-D (99.0% purity), carbendazim (99.0% purity), thiabendazole (98.3% purity), iprodione (99.5% purity), and prochloraz (99.0% purity) were obtained from Dr. Ehrenstorfer (LGC Standards, Augsburg, Germany). HPLC-grade acetonitrile and methanol were purchased from Honeywell International Inc. (Morris Plains, NJ, USA). Analytical-grade acetonitrile, hydrochloric acid (HCl), sodium chloride (NaCl), and anhydrous magnesium sulfate (anhydrous MgSO_4_) for pesticide residue analyses were obtained from Beijing Chemical Reagent Company (Beijing, China). Primary secondary amine (PSA, 40 μm), octadecylsilane (C18, 40 μm), and graphitized carbon black (GCB, 400 mesh) sorbents were purchased from Agela Technologies Inc. (Tianjin, China). Ultra-pure water was obtained from Wahaha Group Co., Ltd. (Hangzhou, China).

### UHPLC–MS/MS analysis

Chromatographic separation was carried out using an Agilent 1290 LC system (Agilent Technologies, Santa Clara, CA) consisting of a four-channel on-line degasser, a standard binary pump, and an Agilent Poroshell120 EC-C18 column (2.1 × 50 mm, 2.7 μm particle size). The mobile phase consisted of ultra-pure water (eluent A) and methanol (eluent B). The gradient elution program was 10% B at injection time, linear increase to 50% B in 1.0 min, further increase to 95% B in 1.5 min, and then maintain for 4.4 min before returning to the initial conditions of 10% B (90% A) in 0.1 min. The flow rate was 0.4 mL min^−1^, and all compounds were eluted within 5.0 min. The temperature of the sample vial holder was set at 5 °C and the column temperature was maintained at 40 °C to decrease viscosity. The injected volume was 1 μL.

An Agilent 6495 triple quadrupole mass spectrometer (Agilent Technologies, Santa Clara, CA, USA) equipped with a conventional ESI source was used to quantify the five compounds of interest. Nitrogen (99.95%) and argon (99.99%) were used as the nebulizer gas and the collision gas, respectively, and the pressure in the T-Wave cell was 3.2 × 10^−5^ MPa. The positive and negative ionization switching modes and MRM were used for the detection of the five compounds, and the MS/MS conditions were optimized for the target compounds. The conditions were typically as follows: source temperature, 200 °C; capillary voltage, 3.0 kV; and desolvation temperature, 370 °C. A cone gas flow of 50 L h^−1^ and a desolvation gas flow of 600 L h^−1^ were used. Infusion experiments were conducted for each compound to optimize the intensity in both positive and negative ionization modes. All other MS parameters were optimized individually for each target compound, and the optimized parameters are listed in Supplementary Table 1. MassHunter software (Agilent, Santa Clara, CA, USA) was used to collect and analyze the data.

### Sample preparation

The QuEChERS procedure is the combination of an extraction step for pesticides in fruits and vegetables and a cleanup step that removes sugars, lipids, and organic acids. And some modifications to the original QuEChERS method have been introduced to ensure efficient extraction of pH-dependent compounds in the vegetables and fruits. Initially, each chopped and homogenized sample (20.0 g) was placed in a 50 mL centrifuge tube, then a mixture of 20.0 mL of acetonitrile:toluene (1:1, v/v, containing 0.25% HCl) was added, and the sample was vortexed for 3 min. Subsequently, 5.0 g of NaCl was added, the tubes were immediately vortexed intensively for 2 min, and then centrifuged at 5000 r min^−1^ for 5 min. Next, 0.1 mL of the upper layer was transferred into a single-use centrifuge tube, diluted with 0.9 mL of methanol, and filtered through a 0.22 μm nylon syringe filter prior to UHPLC–MS/MS injection.

### Validation procedure

Linearity, recovery, precision (as repeatability and reproducibility, relative standard deviation (RSD)), matrix effects, limit of quantification (LOQ), and measurement uncertainty were investigated to determine the accuracy and precision of the analytical method, as described by SANCO/12571/2013^38^. Quantification and performance were determined by comparison with the peak areas of matrix-matched standard solutions. The linearity was analyzed in solvent and matrix without and with 10-fold dilution, using matrix-matched calibration curves with concentration gradients of 0.1, 1, 5, 10, 50, 100, and 200 μg/L for 2,4-D, carbendazim, thiabendazole, and prochloraz, and 0.25, 1, 5, 10, 50, 100, and 200 μg/L for iprodione. The ME (matrix effect) was examined using the following equation:


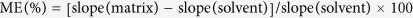


where slope (matrix) and slope (solvent) are obtained from the calibration curves^42^. To study the performance of the method with a reduced matrix effect, solutions without dilution of the blank extracts and with 10-fold dilution of the blank extracts were prepared.

Matrix-matched calibration curves were used to correct for ion suppression/enhancement effects. As a result, the recoveries were analyzed at three levels: LOQ, 10 × LOQ, and 100 × LOQ. The LOQ was set as the minimum concentration that can be quantified with acceptable accuracy and precision^38^.

### Experimental design

An experimental P–B design can provide important information about each variable to allow screening of the main variables that affect the extraction recovery with relatively few experiments^26^,^43^. The five factors or independent variables (X_1_ to X_5_) considered in this study represent the extraction solution composition, HCl percentage, PSA amount, C18 amount, and GCB amount, respectively. All variables were investigated at two levels designated as +1 (high) and −1 (low). Supplementary Table 2 shows the levels of each factor used in the experimental design. The design also includes three central points to estimate the experimental error (pure error)^44^.

Then, the significant factors, such as GCB amount, percentage of HCl, and percentage of toluene, were optimized by using a CCD, and a quadratic model between the dependent and independent variables was built. CCD is one of the most popular response-surface designs used to fit quadratic models, and was first described by Box and Wilson^45^. To fit quadratic polynomials, CCD combines a 2^f^ factorial design with additional points (star points) and at least one point at the center of the experimental region to obtain properties such as rotatability or orthogonality^46^. Subsequently, the specific values of the three most significant variables were identified using DF, which can convert multiple responses into a single response, as follows^30^,^47^.


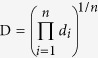


where *n* is the number of responses and *d*_*i*_ is the partial desirability function of each response. The experimental designs were carried out and the results were evaluated using StatSoft Statistica 10.0 (StatSoft, Tulsa, OK, USA).

## Additional Information

**How to cite this article**: Li, M. *et al*. Chemometric-assisted QuEChERS extraction method for post-harvest pesticide determination in fruits and vegetables. *Sci. Rep.* **7**, 42489; doi: 10.1038/srep42489 (2017).

**Publisher's note:** Springer Nature remains neutral with regard to jurisdictional claims in published maps and institutional affiliations.

## Supplementary Material

Supplementary Information

## Figures and Tables

**Table 1:** Linear regression parameters and recoveries for 2,4-D, carbendazim, thiabendazole, iprodione, and prochloraz in various matrices.

Pesticides			Regression equation^*^	R^2^	ME (%)^&^	LOQ^#^ (μg/kg)	Spiked level								
							LOQ			10 × LOQ			100 × LOQ		
	Matrix						Recovery	RSD^a^	RSD^b^	Recovery	RSD^a^	RSD^b^	Recovery	RSD^a^	RSD^b^
2,4-D	Methanol		y = 1991.1x + 325.61	0.9995	−	−	−	−	−	−	−	−	−	−	−
	Citrus	ND^@^	y = 3175.9x − 158.4	0.9996	+60	1.0	91.9	1.8	4.4	96.5	1.9	3.1	98.6	3.6	7.6
		D10^Δ^	y = 2562.4x + 56054	0.9988	+29										
	Apple	ND	y = 2190x + 5653.1	0.9984	+10	1.0	89.2	2.1	6.2	92.1	2.3	1.9	96.6	4.1	6.4
		D10	y = 1923.4x − 447.11	0.9989	−3										
	Mango	ND	y = 2847.7x + 3979.9	0.9965	+43	1.0	89.4	11.7	2.9	87.7	8.4	14.9	93.5	5.1	8.5
		D10	y = 2038.9x − 1135.1	0.9957	+17										
	Lychee	ND	y = 1193.1x − 1987.2	0.9995	−40	1.0	70.6	4.3	5.9	79.2	4.2	2.5	76.5	5.1	7.6
		D10	y = 1551.9x + 10058	0.9985	−22										
	Tomato	ND	y = 1592.8x + 8706.1	0.9988	−21	1.0	71.3	4.5	8.7	78.1	5.2	1.2	72.2	3.7	9.4
		D10	y = 1510.7x + 261.61	0.9974	−24										
	Cucumber	ND	y = 2449.5x + 18404.2	0.9974	+23	1.0	90.6	11.1	4.7	94.9	5.6	12.7	95.8	2.5	7.5
		D10	y = 2035.4x − 1566	0.9962	+2										
	Green pepper	ND	y = 1353.9x − 1499.5	0.9991	−32	1.0	73.5	4.4	6.8	76.4	3.6	3.8	76.1	6.7	10.3
		D10	y = 1602.5x + 269.23	0.9982	−20										
	Eggplant	ND	y = 1164x − 1134.1	0.9995	−42	1.0	79.8	4.1	9.3	78.9	2.5	5.0	73.9	3.2	7.2
		D10	y = 1436x − 390.65	0.9975	−28										
Carbendazim	Methanol		y = 176731x + 99055	0.9998	−	−	−	−	−	−	−	−	−	−	−
	Citrus	ND	y = 268161.8x + 4949.8	0.9968	+52	1.0	86.9	1.2	10.1	81.8	0.6	4.5	74.9	4.8	7.1
		D10	y = 223223x + 27773	0.9991	+26										
	Apple	ND	y = 236215.6x + 2005.6	0.9992	+34	1.0	105.5	1.5	5.5	100.5	1.3	6.0	104.7	1.5	2.2
		D10	y = 190466x − 15186	0.9956	+8										
	Mango	ND	y = 229750.3x − 453.5	0.9998	+30	1.0	84.9	2.8	6.7	83.7	1.4	10.4	82.9	1.1	10.6
		D10	y = 217618x + 200102	0.9985	+23										
	Lychee	ND	y = 128246.2x + 9190.9	0.9979	−27	1.0	75.9	2.3	4.9	84.0	3.8	7.4	102.7	1.9	7.8
		D10	y = 136591x − 2053.7	0.9965	−23										
	Tomato	ND	y = 215811.8x − 5433.4	0.9994	+22	1.0	79.6	1.6	5.3	84.8	1.5	1.8	86.3	1.1	6.3
		D10	y = 208961x − 78290	0.9981	+18										
	Cucumber	ND	y = 266631.1x + 4290	0.9989	+51	1.0	85.6	1.3	8.2	88.9	1.0	10.2	84.4	1.4	7.7
		D10	y = 218324x − 79646	0.9974	+24										
	Green pepper	ND	y = 226515.7x + 4113.7	0.9994	+28	1.0	83.8	1.1	1.5	89.4	2.1	1.0	85.6	2.8	11.6
		D10	y = 193792x − 39174	0.9986	+10										
	Eggplant	ND	y = 243354.1x + 1266.9	0.9959	+38	1.0	101.7	8.9	3.6	107.0	6.5	4.3	96.8	2.4	3.8
		D10	y = 194680x − 105142	0.9987	+10										
Thiabendazole	Methanol		*y* = 122380*x* + 15957	0.9993	−	−	−	−	−	−	−	−	−	−	−
	Citrus	ND	y = 196255.6x − 953.8	0.9999	+60	1.0	107.4	4.5	5.9	104.6	0.9	3.5	86.1	10.9	4.1
		D10	*y* = 160230*x* + 16940	0.9990	+31										
	Apple	ND	y = 126499x + 1470.3	0.9992	+3	1.0	78.2	6.1	2.5	87.8	4.4	3.7	102.2	4.1	14.6
		D10	*y* = 118125*x* + 20244	0.9979	−3										
	Mango	ND	y = 104799.2x + 664.2	0.9988	−14	1.0	96.3	11.9	8.7	109.2	7.7	5.2	106.4	5.8	9.9
		D10	*y* = 100496*x* − 9439.5	0.9976	−18										
	Lychee	ND	y = 59966x − 633.4	0.9996	−51	1.0	102.8	6.6	13.9	107.5	4.4	5.2	108.2	10.2	8.4
		D10	*y* = 71458*x* − 21003	0.9986	−42										
	Tomato	ND	y = 142960.8x + 1786.3	0.9978	+17	1.0	81.8	9.8	5.8	92.3	6.0	12.8	96.4	9.4	10.2
		D10	*y* = 129274*x* − 25846	0.9983	+6										
	Cucumber	ND	y = 134170.4x + 300.1	0.9993	+10	1.0	81.8	7.5	4.6	99.8	4.5	2.9	101.0	5.1	1.6
		D10	*y* = 130301*x* − 20946	0.9999	+6										
	Greenpepper	ND	y = 89537.4x − 654.9	0.9985	−27	1.0	97.3	7.7	13.8	113.9	5.8	3.9	108.7	10.8	10.2
		D10	*y* = 108280*x* − 23063	0.9982	−12										
	Eggplant	ND	y = 89113.6x − 934.8	0.9942	−27	1.0	97.1	4.2	3.1	107.7	3.8	5.9	91.5	1.5	6.3
		D10	*y* = 110845*x* − 7371.8	0.9994	−9										
Iprodione	Methanol		*y* = 262.10*x* + 42.36	0.9994	−	−	−	−	−	−	−	−	−	−	−
	Citrus	ND	y = 173.4x + 20.3	0.9981	−34	1.5	80.3	3.8	1.2	96.9	11.5	4.7	94.9	3.5	11.5
		D10	*y* = 224.26*x* − 206.81	0.9991	−14										
	Apple	ND	y = 214.4x − 159.8	0.9974	−18	1.5	89.7	5.9	1.5	86.5	4.8	1.1	92.4	3.2	6.2
		D10	*y* = 211.76*x* − 142.7	0.9981	−19										
	Mango	ND	y = 154.9x + 1665.4	0.9989	−41	1.5	70.4	7.8	11.9	80.6	4.3	12.6	88.7	8.2	4.5
		D10	*y* = 194.12*x* − 286.19	0.9993	−26										
	Lychee	ND	y = 119.8x − 18.8	0.9997	−54	1.5	79.6	3.2	13.8	90.2	3.4	10.3	95.1	4.9	3.7
		D10	*y* = 215.67*x* + 304.49	0.9994	−18										
	Tomato	ND	y = 148.5x + 190.35	0.9975	−43	1.5	70.6	11.4	14.8	77.1	8.9	4.1	83.4	4.7	6.2
		D10	*y* = 203.24*x* + 183.04	0.9989	−22										
	Cucumber	ND	y = 335.4x + 73.08	0.9963	+28	1.5	92.2	3.9	9.5	97.3	9.3	4.8	109.8	4.8	8.3
		D10	*y* = 305.57*x* + 151.04	0.9989	+17										
	Green pepper	ND	y = 217.3x + 222.6	0.9994	−17	1.5	80.4	7.6	6.2	87.6	8.7	3.5	95.5	5.4	7.9
		D10	*y* = 240.43*x* + 127.7	0.9998	−8										
	Eggplant	ND	y = 121.05x − 343.2	0.9987	−54	1.5	71.0	11.3	16.9	77.5	5.6	9.8	82.2	5.8	9.0
		D10	*y* = 141.56*x* + 148.84	0.9988	−46										
Prochloraz	Methanol		*y* = 14490.3*x* + 7062.4	0.9995	−	−	−	−	−	−	−	−	−	−	−
	Citrus	ND	y = 18492.5x + 6884.3	0.9998	+28	1.0	112.0	10.9	2.8	82.3	5.4	1.3	107.9	10.1	7.2
		D10	*y* = 16959.8*x* + 10809	0.9990	+17										
	Apple	ND	y = 9118.8x−433.8	0.9995	−37	1.0	71.9	9.6	8.3	75.1	5.2	11.9	78.2	4.7	7.6
		D10	*y* = 10237.7*x* − 1370.6	0.9980	−29										
	Mango	ND	y = 19541.9x + 2436.5	0.9985	+35	1.0	98.5	10.7	7.4	109.8	11.2	2.9	105.4	6.9	6.7
		D10	*y* = 20050*x* − 2753.5	0.9984	+38										
	Lychee	ND	y = 7976.6x + 2117.7	0.994	−45	1.0	86.7	1.8	3.3	89.0	9.2	5.0	85.6	7.5	4.2
		D10	*y* = 11632*x* + 34578	0.9991	−20										
	Tomato	ND	y = 18652.4x + 22843.5	0.9982	+29	1.0	84.5	3.8	6.8	88.3	1.8	9.5	83.5	7.4	12.4
		D10	*y* = 16274*x* − 7563.4	0.9988	+12										
	Cucumber	ND	y = 18557.7x + 2467.2	0.9990	+28	1.0	87.7	2.9	4.3	90.0	3.2	2.6	99.6	2.9	6.9
		D10	*y* = 18532*x* + 18399	0.9983	+28										
	Greenpe pper	ND	y = 19996x − 21135.3	0.9995	+38	1.0	83.0	5.0	8.7	85.7	2.6	12.5	88.9	7.0	5.5
		D10	*y* = 17957*x* + 2000.7	0.9998	+24										
	Eggplant	ND	y = 8684.1x + 4553.8	0.9974	−40	1.0	86.6	7.1	1.5	89.6	3.3	4.3	87.7	3.8	7.2
		D10	*y* = 12810*x* − 1936	0.9996	−12										

^@^Matrix with no dilution. ^Δ^Matrix with 10 times dilution. *The calibration range was 0.1–200 μg/kg for all preservative except 0.25–200 μg/kg for iprodione; ^&^Matrix effect; ^#^Limits of quantification.

**Table 2:** Uncertainty (*U*_c_) and expanded uncertainty (*U*_exp_) in different matrices for 2,4-D, carbendazim, thiabendazole, and prochloraz at 1.0–100 μg/kg and iprodione at 1.5–150 μg/kg.

Uncertainty	2,4-D	Carbendazim	Thiabendazole	Iprodione	Prochloraz
*U*_1_	0.0015	0.0015	0.0015	0.0021	0.0015
*U*_2_	0.0302	0.0205	0.0232	0.0027	0.0285
*U*_3_	0.0035	0.0048	0.0045	0.0027	0.0059
*U*_4_	0.0298	0.0213	0.0303	0.0371	0.0306
*U*_c_	0.0425	0.0299	0.0385	0.0374	0.0423
*U*_exp_ (%)	8.5	5.9	7.7	7.5	8.4

**Table 3:** Concentrations of 2,4-D, carbendazim, thiabendazole, iprodione, and prochloraz in vegetable and fruit samples obtained from Beijing markets.

Samples	Number of samples	Positive sample ratio^a^	Concentration (μg/kg)^b^				
			2,4-D	Carbendazim	Thiabendazole	Iprodione	Prochloraz
Citrus	20	6 (30%)	2 (2.6/8.5)	3 (1.5/5.5/12.8)	1 (2.4)	<LOD	<LOD
Apple	10	2 (20%)	<LOD	2 (3.5/7.2)	<LOD	<LOD	<LOD
Mango	10	0	<LOD	<LOD	<LOD	<LOD	<LOD
Lychee	20	1 (5%)	<LOD	1 (3.1)	<LOD	<LOD	<LOD
Tomato	5	1 (20%)	<LOD	1 (10.6)	<LOD	<LOD	<LOD
Cucumber	5	0	<LOD	<LOD	<LOD	<LOD	<LOD
Green pepper	10	0	<LOD	<LOD	<LOD	<LOD	<LOD
Eggplant	5	0	<LOD	<LOD	<LOD	<LOD	<LOD

^a^Number of positive sample (positive sample ratio).

^b^Number of detectable sample (concentration of pesticide).
