# Supplementary material for: Chemometric-assisted QuEChERS extraction method for post-harvest pesticide determination in fruits and vegetables
Source: Sci Rep. 2017 Feb 22;7:42489. doi: 10.1038/srep42489 (PMC5320482; doi:10.1038/srep42489)
Supplement: Supplementary Information [file srep42489-s1.pdf]

[Supplementary material] including 1 figure and 3 tables

Chemometric-assisted QuEChERS extraction method for post-harvest pesticide determination in fruits and vegetables

Minmin Li [a,b,1], Chao Dai[a,1], Fengzhong Wang[a], Zhiqiang Kong[a,b,]*, Yan He [a], Yatao Huang [a], Bei Fan[a,]*

[1] These authors contributed equally to this paper.

[a] Institute of Food Science and Technology, Chinese Academy of Agricultural Sciences/Key Laboratory of Agro-Products Processing/Laboratory of Agro-Products Quality Safety Risk Assessment, Ministry of Agriculture, Beijing 100193, P. R. China

[b] Functional and Evolutionary Entomology, Gembloux Agro-Bio-Tech, University of Liège, Passage des Déportés 2, 5030 Gembloux, Belgium

*Corresponding author. Tel.: +86 10 62813566; fax: +86 10 62813566
E-mail address: kongzhiqiang@caas.cn
*Corresponding author. Tel.: +86 10 62810295; fax: +86 10 62810295
E-mail address: fanbeicaas@163.com

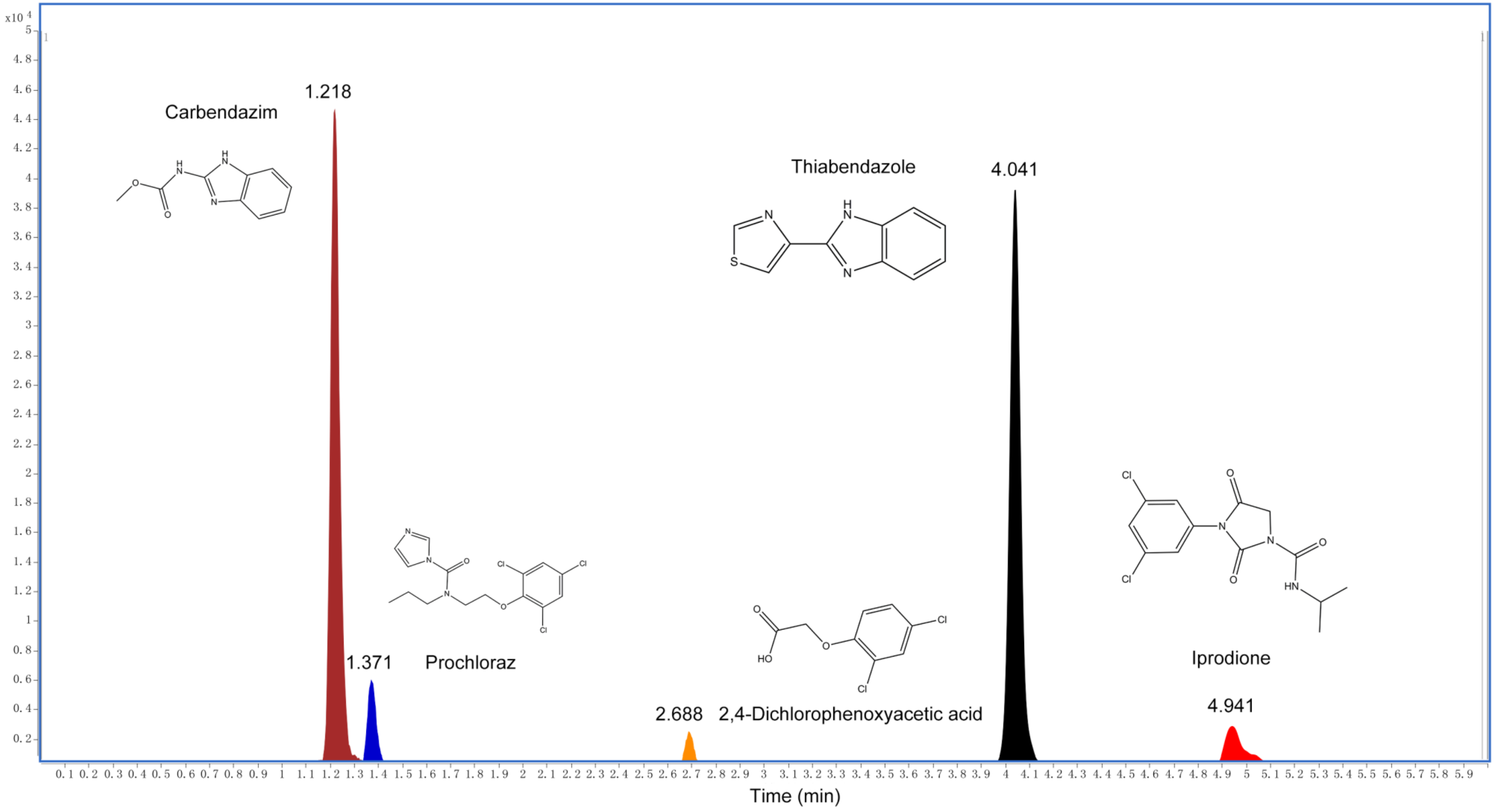

Figure 1. Structures of the five standard pesticides and typical total ion current (TIC) chromatograms at concentrations of 50 μg/L.

Table S1. Optimized MS parameters for determination of 2,4-D, carbendazim, thiabendazole, iprodione, and prochloraz using UHPLC-MS/MS.

Compounds	Molecular weight	Precursor ion (m/z)	Product ion (m/z)	Fragmentor voltage (V)	Collision energy (eV)	Retention time (min)	ESI mode
2,4-D	221.04	220	163	380	20	2.72	Negative
		219	161	380	10		
Carbendazim	191.19	192.1	160	380	20	1.22	Positive
		192.1	132.1	380	40		
Thiabendazole	201.25	202.2	175.1	380	25	1.36	Positive
		202.2	131.1	380	35		
Iprodione	330.17	331.2	245	380	10	3.82	Positive
		331.2	56.1	380	45		
Prochloraz	376.67	377.6	308	380	5	4.01	Positive
		377.6	70.1	380	20		

Table S2. Experimental factors and levels of the Plackett–Burman design.

Factor	Unit	Level		
		Low(-1)	Central(0)	High(+1)
(X_1) Toluene percentage	%	0	50	100
(X_2) HCl percentage	%	0	0.25	0.5
(X_3) PSA amount	mg	0	25	50
(X_4) C18 amount	mg	0	10	20
(X_5) GCB amount	mg	0	10	20

Table S3. Regression analysis results for extraction recovery of 2,4-D, carbendazim, thiabendazole, iprodione, and prochloraz.

Source	2,4-D		Carbendazim		Thiabendazole		Iprodione		Prochloraz	
	Coefficient	*p*-value	Coefficient	*p*-value	Coefficient	*p*-value	Coefficient	*p*-value	Coefficient	*p*-value
X_0	54.57	-	70.51	-	76.06	-	75.13	-	67.39	-
X_1	0.73	0.0251	0.40	0.0193	0.93	0.0334	0.13	0.1483	-0.57	0.0278
X_2	-14.06	0.0246	53.43	0.1216	25.18	0.0251	-50.17	0.0471	92.50	0.0419
X_3	2.22	0.0096	-2.21	0.0498	1.57	0.2147	-3.09	0.0082	-2.19	0.0196
X_1^2	-0.01	0.0313	-0.23	0.0670	-0.19	0.0249	0.01	0.8642	-0.15	0.0329
X_2^2	19.93	0.0446	-10.66	0.1073	54.76	0.4754	-75.94	0.0648	-13.41	0.0373
X_3^2	-0.05	0.1839	0.55	0.1396	0.18	0.9861	0.14	0.0078	0.04	0.1994
X_1X_2	-0.34	0.0361	0.16	0.2737	0.05	0.8091	-0.19	0.0841	-0.25	0.1338
X_1X_3	-0.11	0.7764	-0.05	0.2028	-0.02	0.7373	0.43	0.1019	0.01	0.7963
X_2X_3	1.60	0.0452	0.40	0.5329	1.25	0.3029	-0.85	0.1001	-0.05	0.9308
